# SMA Human iPSC-Derived Motor Neurons Show Perturbed Differentiation and Reduced miR-335-5p Expression

**DOI:** 10.3390/ijms17081231

**Published:** 2016-07-30

**Authors:** Michela Murdocca, Silvia Anna Ciafrè, Paola Spitalieri, Rosa Valentina Talarico, Massimo Sanchez, Giuseppe Novelli, Federica Sangiuolo

**Affiliations:** 1 Department of Biomedicine and Prevention, University of Rome Tor Vergata, Via Montpellier, 1, 00133 Rome, Italy; miky.murdi@hotmail.it (M.M.); paola.spitalieri@uniroma2.it (P.S.); valentinatalarico@hotmail.it (R.V.T.); novelli@med.uniroma2.it (G.N.); 2 Department of Cell Biology and Neurosciences, Istituto Superiore di Sanità, 00161 Rome, Italy; massimo.sanchez@iss.it

**Keywords:** SMA, early motor neuron, miRNA, hiPSCs

## Abstract

Spinal Muscular Atrophy (SMA) is a neuromuscular disease caused by mutations in the Survival Motor Neuron 1 gene, resulting in very low levels of functional Survival of Motor Neuron (SMN) protein. SMA human induced Pluripotent Stem Cells (hiPSCs) represent a useful and valid model for the study of the disorder, as they provide in vitro the target cells. MicroRNAs (miRNAs) are often reported as playing a key role in regulating neuronal differentiation and fate specification. In this study SMA hiPSCs have been differentiated towards early motor neurons and their molecular and immunocytochemical profile were compared to those of wild type cells. Cell cycle proliferation was also evaluated by fluorescence-activated cell sorting (FACS). SMA hiPSCs showed an increased proliferation rate and also higher levels of stem cell markers. Moreover; when differentiated towards early motor neurons they expressed lower levels of NCAM and MN specific markers. The expression of miR-335-5p; already identified to control self-renewal or differentiation of mouse embryonic stem cells (mESCs); resulted to be reduced during the early steps of differentiation of SMA hiPSCs compared to wild type cells. These results suggest that we should speculate a role of this miRNA both in stemness characteristic and in differentiation efficiency of these cells.

## 1. Introduction

Spinal Muscular Atrophy (SMA) is a neuromuscular disease, recessively inherited, representing the primary genetic reason of infantile mortality. SMA is caused by mutations in the survival motor neuron 1 (*SMN1*; MIM#600354) gene, resulting in very low levels of functional SMN protein [1,2]. The disease is characterized by the degeneration of lower α-motor neurons (MN), progressive muscle weakness and paralysis [3]. Although SMA is caused by reduced levels of a ubiquitous protein, the higher sensitivity of motor neurons to SMN deficiency still represents an unsolved paradox. Moreover, no effective treatment has been developed for this motor neuron disease.

In recent years, a great interest has been growing for induced pluripotent stem cells (iPSCs) as a model for the study of genetic diseases, and in particular for those characterized by the inaccessibility of the disease target cells in patients. SMA, affecting MNs, is one of these.

Human iPSCs from SMA patients have already been described [4,5], closely resembling an in vitro model for the pathology. Ebert and collaborators showed that hiPSCs from type I SMA patients are able to differentiate into motor neurons lacking SMN1 expression, and undergo a selective death over time.

Recent findings have placed microRNAs (miRNAs) in the midst of gene regulatory networks involved in neural induction, neuronal differentiation and fate specification [6].

We have recently shown that miR-335-5p, already identified to control self-renewal or differentiation of mESCs [7], is differentially expressed in SMN∆7 SMA versus wild type (WT) neural progenitor cells derived from E13.5 mice spinal cords, and probably correlated to the increased proliferation activity observed in SMA cells [8].

In this study, we analyze the molecular and phenotypic characteristics, in terms of gene expression and cell cycle proliferation, of SMA and wild type hiPSCs during their differentiation towards early motor neurons, and we confirm the under expression of miR-335-5p in SMA cells, associated to a reduced expression of early MN markers.

## 2. Results

**Figure 1:**
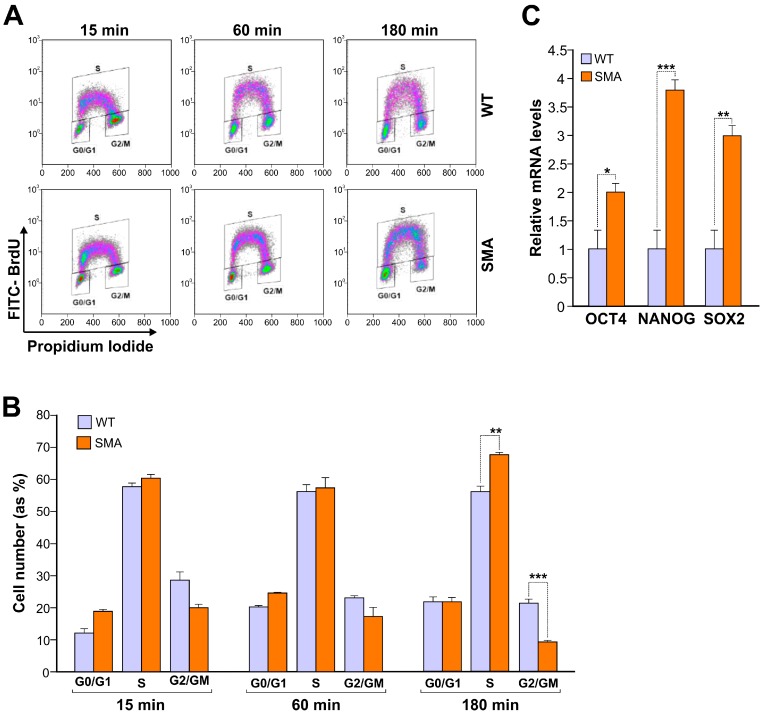
Spinal Muscular Atrophy (SMA) human induced Pluripotent Stem Cells (hiPSCs) show an increased proliferation rate and expression levels of stem cell markers compared to wild type (WT) ones. (**A**) Flow cytometry analysis of bromodeoxyuridine (BrdU)-positive cells of SMA- and WT-derived hiPSCs incubated with BrdU for 15, 60 and 180 min. Representative dot plots of cell cycle reporting the BrdU-positive cells are shown. Three regions have been drawn in each dot plot to identify G0/G1, S and G2/M subpopulations; (**B**) The column bar graph reports the percent of cells in G0/G1, S and G2/M phases of SMA- and WT-derived hiPSCs after 15, 60 and 180 min of BrdU incubation. The results (mean ± standard deviation) are representative of three independent experiments (** *p* < 0.01, *** *p* < 0.001); (**C**) Real time-qPCR analysis of OCT4, NANOG and SOX2 in SMA and WT-hiPSCs using the expression of WT sample as the reference. The data were normalized to 5S ribosomal RNA expression. Data are representative of three independent replicates; values represent mean ± SD; *** *p* < 0.001, ** *p* < 0.01, * *p* < 0.05.

As we previously described, SMA neural progenitor cells obtained from spinal cords of SMA embryo mice showed an increased cell proliferation rate compared to wild type ones. Thus, we decided to characterize hiPSCs by evaluating the progression along the cell cycle of SMA and WT derived hiPSCs after 15, 60 and 180 min of BrdU incubation (Figure 1A).

**Figure 2:**
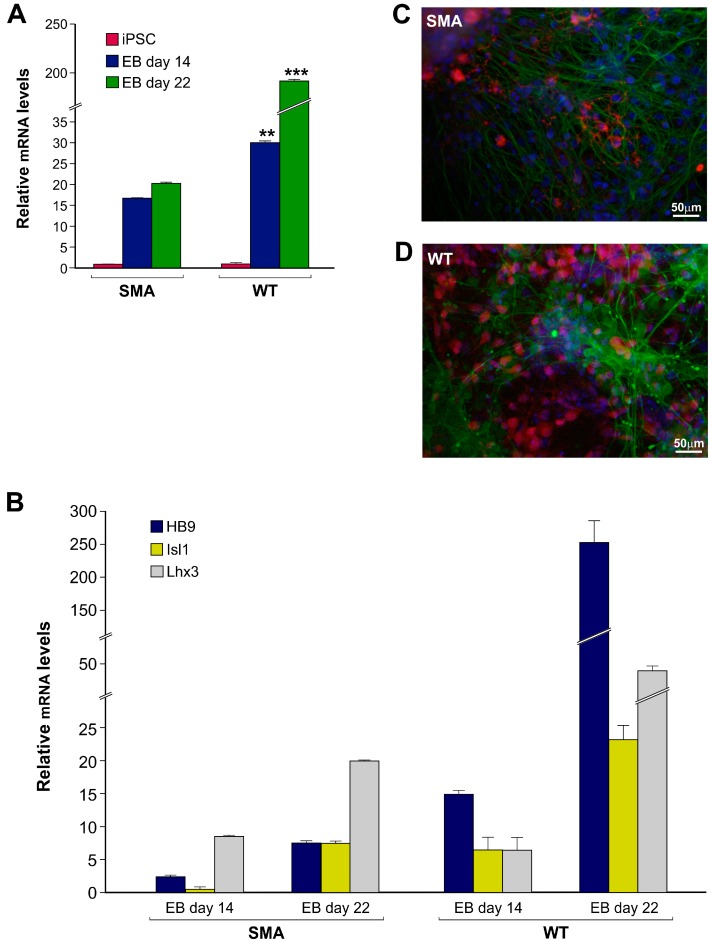
Differential expression of Neural Cell Adhesion Molecule (NCAM) and MN-specific transcription factors along differentiation of SMA and WT hiPSCs. (**A**) RT-qPCR analysis of NCAM expression in WT- and SMA-derived α-motor neurons (MNs) after 14 and 22 days using hiPSCs as a reference. The data are normalized to 5S ribosomal RNA and the expression in hiPSCs was set as =1 in each genotype. Data are representative of three independent replicates; values represent mean ± SD; when comparing WT versus SMA at each time point, *** *p* < 0.001, ** *p* < 0.01; (**B**) The induction of MN differentiation results in transcriptional boost of Isl1, Lhx3 and HB9 in WT cells, while it is lower in SMA ones. The data are normalized to 5S ribosomal RNA and the expression levels in hiPSCs were used as a reference in each genotype. Data are representative of three independent replicates; values represent mean ± SD; (**C**,**D**) Representative immunofluorescence images of in WT- and SMA-derived MNs after 22 days of differentiation, expressing β-III tubulin (TUJ1, green) and LIM3 (red). DAPI nuclear staining is in blue. Scale bars, 50 µm.

Analysis has been performed by flow cytometry in BrdU labelled cells. Although the results show an elevated proliferation proficiency of both SMA- and WT-derived hiPSCs, the former are characterized by a significantly increased number of cells entered in S phase (67.91% ± 0.66% vs. 56.38% ± 1.68% at 180 min), combined with significantly reduced number of cells in G2/M phase (9.57% ± 0.08% vs. 21.60% ± 0.73%), most likely due to a faster exit from mitosis (Figure 1B). In parallel, we analyzed by RT-qPCR the expression of three transcription regulators, NANOG, OCT4 and SOX2, essential for maintaining self-renewal of stem cells. As shown in Figure 1C, the SMA hiPSCs express significantly higher levels of all transcripts compared to wild type hiPSCs (Figure 1C). These data suggest a potential correlation between the observed increase of proliferation rate and the higher expression of “stemness” transcription regulators in SMA hiPSCs. Thus, to better clarify if these aspects could have any consequences on hiPSCs differentiation capacity, cells were induced to embryoid body (EB) formation and specifically committed to the ectodermal lineage. While the expression of stem cell markers (OCT4, NANOG and SOX2) equally decreased in both genotypes (data not shown), RT-qPCR analysis (Figure 2A) showed a statistically significant increase of the ectodermal marker “Neural Cell Adhesion Molecule” (NCAM) expression in WT hiPSCs, strongly evident at 22 days after EB adhesion (*** *p* < 0.001).

**Figure 3:**
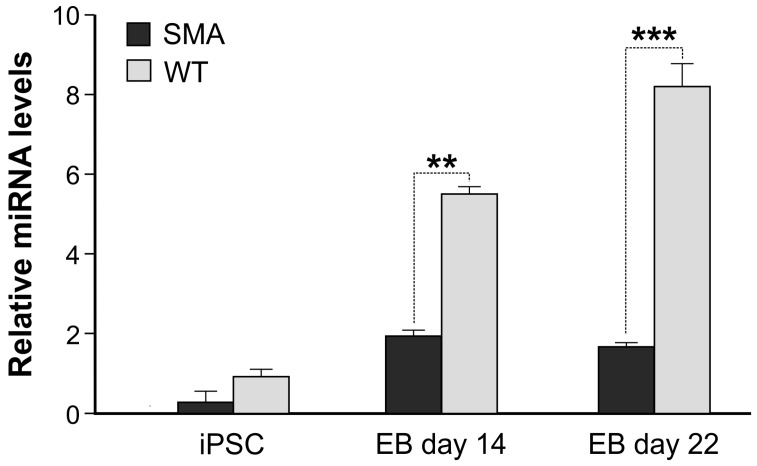
Early MN derived from SMA hiPSCs express reduced levels of miR-335-5p. RT-qPCR of miR-335-5p both in hiPSCs and in early MNs (EB day 14 and EB day 22). The data were normalized to the expression of snRNA U6. Data are representative of three independent replicates; values represent mean ± SD; *** *p* < 0.001, ** *p* < 0.01.

Conversely, only a slight and not significant increase was observed in SMA hiPSCs over time. As the Sonic hedgehog (Shh)-induced transcriptional pathway is critical for the proper induction of early MN differentiation [9], we measured the expression levels of Shh-related MN markers at the same time points during EB differentiation. The expression levels of Lhx3, Isl1 and HB9 in SMA-hiPSC derived MNs strongly increase during the differentiation process until day 22 (*p* < 0.05) (Figure 2B). The same pattern was observed in wild type cells but the expression resulted to be more strongly incremented, especially following 22 days of differentiation (*p* < 0.01) (Figure 2B). In particular HB9 marker, involved in motor neuron specification and maturation [10], showed an evident boost of expression in WT cells (*p* < 0.001), though unparalleled in SMA cells (Figure 2B). Immunocytochemical analysis performed on hiPSC-derived MNs showed a strong positivity to both LIM3 and TUJ1 markers, also in this case the percentage of LIM3 positive cells resulted to be lower in SMA hiPSC-induced MNs supporting the molecular data (Figure 2C,D). Taken together, these results suggest that SMA hiPSCs demonstrate that they differentiate less efficiently into ectodermal derived cells, and specifically into the target cells of the disease. To further corroborate these data and considering our previous results [8], we also evaluated the expression of miR-335-5p in hiPSCs and followed it up along the in vitro differentiation steps from hiPSCs to EBs, at the same time points where we observed the differential expression of early MN markers. RT-qPCR revealed that, while miR-335-5p expression does not differ between SMA and WT hiPSCs, a significant difference is evident at both 14 and 22 days of differentiation. In particular, miR-335-5p results downregulated more than two-fold in SMA samples compared to WT ones after 14 days of differentiation, and even more strongly reduced after 22 days (Figure 3). This suggests a possible correlation between miR-335-5p expression and the ability of hiPSCs to enter the early stages of differentiation towards motor neurons.

## 3. Discussion

One of the most important aspects of hiPSC technology is the possibility of modelling human diseases using patient-derived reprogrammed cells. The recapitulation of the pathological phenotypes using disease-specific hiPSCs that can be differentiated in vitro, sets the basis for studying the aetiopathology of diseases, especially those in which the target cells are really inaccessible [11,12]. In a previous work we described a perturbed pattern of microRNA expression when comparing neural progenitor cells derived from the spinal cords of E13.5 SMA mice to WT. In particular, miR-335-5p, associated with self-renewal, resulted as underexpressed in the SMA model of murine neural precursors. Our present data allow us to take a further step towards the characterization of some molecular events depicting SMA phenotype during the very early stages of differentiation. In fact, not only are we describing human cells, derived from SMA patients, as opposed to the previously studied murine cells, but we are studying them from the undifferentiated state along the early stages of differentiation. We believe that the time points modelled in vitro in our experiments may be important for neuronal differentiation. Our results about the reduced expression of miR-335-5p in SMA cells during differentiation are novel and important, as they indicate a possible role for this miRNA in SMA disease in humans, specifically in cells which are committed towards motor neurons. In fact, human iPSCs before differentiation do not show any differences in miR-335-5p expression, which become evident during EBs differentiation. These observations deserve further deepening in order to unravel if miR-335-5p reduction not only marks the differentiation of SMA neural precursors, but most importantly if it is causally related to their pathological phenotype.

## 4. Materials and Methods

### 4.1. Ethics Statement

This study was conducted according to the principles expressed in the Declaration of Helsinki, and approved by the institutional review board of the Bioethical Committee of Fondazione PTV, Tor Vergata Hospital (prot. 0027655/2013). All patients provided written informed consent for the collection of samples and subsequent analysis.

### 4.2. Cell Culture and Reprogramming to hiPSCs

Chorionic villus sampling (CVSs) were obtained from SMA I high-risk pregnancies or from wild type at the 11th week of gestation, following standard biopsy procedures. Cell culture and hiPSCs reprogramming protocol were previously published [11]. Two independent SMA and wild type hiPS cell preparations were analyzed.

### 4.3. Cytofluorimetry

Flow cytometry analysis on hiPSCs (WT and SMA) were performed as already described [8].

### 4.4. Expression Analyses

For gene expression analyses, total RNA from iPS cells and embryoid bodies (EB) was extracted with TRIzol Reagent (Invitrogen; Life Technologies Corporation, Carlsbad, CA, USA), reverse transcribed with the High-Capacity cDNA Archive kit (Life Technologies Corporation) and used in RT-qPCR, by employing either SYBR Green or TaqMan chemistry (Life Technologies Corporation) and specific primers. The comparative ΔΔ*C*t method was used to quantify relative gene expression levels. All primer sequences for molecular analyses are reported in Table 1.

For miRNA expression, total RNA extracted as reported above, was reverse transcribed using the miR-335 and snRNA U6 Taqman assays (Life Technologies Corporation). The quantitative stem-loop real time polymerase chain reaction (qPCR) was performed according to conditions suggested by Life Technologies.

### 4.5. hiPSCs Differentiation and Immunocytochemical Analysis

HiPSCs were differentiated inMNs according to the protocol described by Amoroso et al. [13]. At day 20, EBs were dissociated with PAPAIN (Worthington, Biochemical Corporation, Lakewood, NJ, USA) and plated onto poly-lysine/laminin-coated 8-well chamber slides (BD Biosciences, Two Oak Park, Bedford, MA, USA). Immunocytochemistry was carried out for the detection of specific neural markers: β-III-tubulin (TUJ1; Abcam, Cambridge, UK; 1:500), and LIM3 (Millipore Corporation, Billerica, MA, USA; 1:250). The cell nucleus was labeled with 4,6-diamidino-2-phenylindole (DAPI; Sigma Aldrich, St. Louis, MO, USA) and examined under a fluorescence microscope. Images were acquired using a Zeiss (Thornwood, NY, USA) Axioplan 2 microscope.

All values provided in the text and figures are means of three independent experiments ± standard deviations (SD). Mean values were compared using the two-tailed Student *t*-test, for independent samples. *p*-Value was considered significant *** *p* < 0.001, ** *p* < 0.01, * *p* < 0.05. FACS Statistical analysis was performed according to paired Student’s *t*-test by using GraphPad Prism Software version 5.03 (GraphPad Software Inc., La Jolla, CA, USA).

## Figures and Tables

**Table 1:** Oligonucleotide sequence.

Primers	Forward (5′-3′)	Reverse (5′-3′)
5S	TCGTCTGATCTCGGAAGCTAAGCA	AAAGCCTACAGCACCCGGTATT
OCT4	AACCTGGAGTTTGTGCCAGGGTTT	TGAACTTCACCTTCCCTCCAACCA
SOX2	AGAAGAGGAGAGAGAAAGAAAGGGAGAGA	GAGAGAGGCAAACTGGAATCAGGATCAAA
NANOG	CCTGAAGACGTGTGAAGATGAG	GCTGATTAGGCTCCAACCATAC
NCAM	ATGGAAACTCTATTAAAGTGAACCTG	TAGACCTCATACTCAGCATTCCAGT
Isl1	GAATGGCATGCGGCATGTTTGA	CGCATTTGATCCCGTACAACCTGA
Lhx3	TCGGACAAGGACAGCGTTCAG	TTTCCGCCAAGGAAGGCTCATCG
HB9	CACCGAGACCCAGGTGAAGATTT	CCCTTCTGTTTCTCCGCTTCCT

OCT4: octamer-binding transcription factor 4; NCAM: Neural Cell Adhesion Molecule; Lhx3: LIM Homeobox 3.
